# Outcome and Oxygenation Response to Airway Pressure Release Ventilation (APRV) Used as a Rescue Strategy for Severe Hypoxemic Respiratory Failure—An Observational Study

**DOI:** 10.3390/jcm15072668

**Published:** 2026-04-01

**Authors:** Harriet Jacobs, Mark Brown, Elizabeth Webb, Olivia Cox, Isis Terrington, Tanvi Dabke, Diana David, Benjamin Eastwood, Ryan Beecham, Michael P. W. Grocott, Ahilanandan Dushianthan

**Affiliations:** 1 Faculty of Medicine, University of Southampton, Southampton SO16 6YD, UK; hj3n22@soton.ac.uk (H.J.); mark.brown@soton.ac.uk (M.B.); a.dushianthan@soton.ac.uk (A.D.); 2 General Intensive Care Unit, University Hospital Southampton NHS Foundation Trust, Southampton SO16 6YD, UK; elizabeth.webb@uhs.nhs.uk (E.W.); olivia.cox@uhs.nhs.uk (O.C.); isis.terrington1@nhs.net (I.T.); tanvidabke1@gmail.com (T.D.); diana.david@uhs.nhs.uk (D.D.); benjamin.eastwood@uhs.nhs.uk (B.E.); ryan.beecham@uhs.nhs.uk (R.B.); 3 NIHR Biomedical Research Centre, University Hospital Southampton and University of Southampton, Southampton SO16 6YD, UK

**Keywords:** mechanical ventilation, APRV, oxygen, intensive care

## Abstract

**Background and objectives:** Airway pressure release ventilation (APRV) is an inverse ratio ventilation often used as a rescue method for patients with severe acute hypoxemic respiratory failure (AHRF). This observational cohort study aims to evaluate the outcomes of APRV for patients with severe AHRF. **Methods:** We conducted a retrospective observational cohort study of patients with AHRF requiring APRV. The primary outcome was early physiological response, defined as the change in PaO_2_/FiO_2_ (P/F ratio) within 6 h of APRV initiation. Secondary outcomes included ICU mortality, duration of mechanical ventilation, and length of stay. **Results:** Between 01/2018 and 09/2024, 152 patients had APRV initiated for >2 h. P/F ratio improved in 64% of patients (responders). Responders had more severe hypoxemia before APRV initiation (P/F ratio 9.8 vs. 10.4 kPa, *p* = 0.05) and showed a greater improvement within 6 h following APRV initiation (18.8 vs. 11.9 kPa, *p* < 0.01). Overall survival to ICU discharge was 56%. There were no differences in ICU survival or liberation from mechanical ventilation between responders and non-responders (log-rank *p* = 0.48 and 0.96). There were also no differences in improvement in oxygenation following APRV or mortality between COVID-19 and non-COVID-19 patients. **Conclusions:** APRV may improve oxygenation in the short term, but this improvement in oxygenation was not associated with improved clinical outcomes in this observational cohort.

## 1. Introduction

Advanced respiratory support for acute hypoxemic respiratory failure (AHRF) is an essential practice in the Intensive Care Unit (ICU). In England, age-standardised rates of invasive mechanical ventilation in adults (IMV) have been recorded at 131 per 100,000 population, with 70% of this population being represented by non-surgical patients [1]. The latest data published by the Intensive Care National Audit and Research Centre (ICNARC) reveal that 42.5% of ICU admissions require advanced respiratory support, representing >86,000 patients annually [2]. While there are several mechanical ventilator modes with variations in pressure/volume controlled/regulated or adaptive modes, lung-protective low tidal volume (6–8 mL/kg IBW) ventilation is commonly adopted when managing patients with hypoxemic respiratory failure from primary lung disease or acute respiratory distress syndrome [3,4].

Airway pressure release ventilation (APRV) is an alternative pressure-controlled, time-cycled inverse I/E ratio mode that uses a prolonged inspiratory pressure (P_high_) for an extended inspiratory time (T_high_) and a brief expiratory release time (T_low_). This facilitates more homogeneous alveolar recruitment while allowing spontaneous breathing [5]. Several small trials have evaluated APRV as a primary mode of ventilation, particularly in patients with acute respiratory distress syndrome (ARDS), and meta-analyses of these studies have suggested potential benefits, including increased ventilator-free days and shorter ICU days [6,7]. However, most of these studies were small and varied considerably in their intervention protocols [7,8,9]. Due to a lack of robust evidence, major ARDS or AHRF guidelines do not make any APRV-specific recommendations [10,11].

In contrast to randomised controlled trials, observational studies used APRV primarily as a rescue measure rather than an initial primary ventilation strategy. In the UK, 55.7% of ICUs use APRV as part of rescue strategies for AHRF, and APRV is rarely used as a “start-up” ventilation mode [12]. Notably, the adoption of APRV has increased over the past decade, likely due to its increased use during the COVID-19 pandemic [13,14,15]. Despite the key differences between RCTs and observational studies, APRV is commonly used as a rescue strategy for severe hypoxemic respiratory failure. While there is evidence that APRV improves oxygenation, it remains unclear whether these physiological effects are associated with meaningful clinical benefits such as improved survival [16,17]. As a result, further real-world evaluation of APRV use and its impact on patient outcomes is warranted. We hypothesised that early improvement in oxygenation following the initiation of APRV may confer improved clinical outcomes in patients with severe hypoxemic respiratory failure treated in routine clinical practice. Therefore, we evaluated the use of APRV in our centre to determine whether improved oxygenation was associated with robust outcome measures such as mortality.

## 2. Materials and Methods

This was a retrospective, observational cohort study conducted in a general ICU based in a tertiary centre in the South of England. All patients ventilated with APRV mode from 1 January 2018 to 30 September 2024 were reviewed for inclusion into the study. The data for non-COVID-19 patients were analysed across the entire study period (1 January 2018 to 30 September 2024), whereas patient data of patients with COVID-19 pneumonia were included from the start of the pandemic (2020 onwards). The study cohort consisted of consecutive ICU patients who met the pre-defined inclusion criteria during the study. All patients who met the inclusion criteria were included in the study. This study was part of a wider study investigating outcomes of critical illness in intensive care (CRIT-CO). CRIT-CO is sponsored by University Hospital Southampton NHS Foundation Trust (RHM CRI 0370) and has approval from the NHS Health Research Authority (HRA, UK: IRAS 232922). All identifiable patient data has been anonymised. This study is compliant with HRA standards, and consent was waived given the retrospective design of the study. The study adhered to Strengthening the Reporting of Observational Studies in Epidemiology (STROBE) guidelines for reporting observational studies [18].

### 2.1. Data Collection

Patients were identified using electronic hospital records. Data were gathered using ICU and hospital clinical notes from the two online platforms used at the trust, MetaVision (iMDsoft, Tel Aviv, Israel) and CHARTS (custom software for University Hospital Southampton NHS Trust, version 35). Through data coding, patients ventilated using APRV mode during this period were identified. Each identified patient was reviewed against the following inclusion/exclusion criteria.

#### 2.1.1. Inclusion Criteria

- Adult patient (>18 years).
- Required ICU admission.
- A documented diagnosis of ARDS, based on the Berlin Definition of ARDS, with a PaO_2_/FiO_2_ ratio (P/F ratio) < 40 kPa (<300 mmHg) at the time of APRV initiation [19].

#### 2.1.2. Exclusion Criteria

- Under <18 years of age.
- APRV use for <2 h.

The study cohort consisted of consecutive ICU patients who met the pre-defined inclusion criteria during the study. All patients who met the inclusion criteria were included in the study. Once inclusion into the study was confirmed, detailed data on demographic variables, including age, sex, BMI, and comorbidities, as well as details of oxygenation, APRV use, and outcomes, were collected. We further collected detailed parameters, including laboratory variables, Charlson Comorbidity Index [20], Acute Physiology Assessment and Chronic Health Evaluation (APACHE) II Score [21], Sequential Organ Failure Assessment (SOFA score) [22], and Chest X-Ray Radiographic Assessment of Lung Edema (CXR RALE) score [23]. Following data collection, patients were categorised according to mortality, response to APRV, and COVID-19 status. All patients were followed up until discharge from hospital. Response to APRV was determined by using the mean P/F ratio for the 6 h pre-intervention compared to the mean P/F ratio for the subsequent 6 h post-intervention, reflecting early physiological response to the intervention. Patients who showed an improvement in P/F ratio following APRV were defined as responders. Patients with a COVID-19-positive diagnosis at ICU discharge were classed as COVID-19 AHRF.

### 2.2. Intervention

All patients were intubated and mechanically ventilated. In our centre, APRV is used as a rescue strategy for patients with severe hypoxemic respiratory failure of all causes. Our standard practice includes deep sedation, paralysis, and trial of prone positioning if there are no contraindications. Prior to APRV initiation, all patients were managed using conventional lung-protective ventilation strategies including volume- or pressure-controlled ventilation with tidal volumes targeting approximately 6–8 mL/kg predicted body weight and appropriate PEEP titration according to the ARDSnet protocol [3]. APRV was introduced as a rescue strategy when severe hypoxemia persisted (P/F of <13.3 kPa) despite optimisation of conventional ventilation. APRV in our ICU was commenced and titrated using a standardised institutional approach based on previously published RCT and guidelines of APRV physiology and implementation [5,24]. Clinicians followed this framework when selecting P_high_, P_low_, T_high_, and T_low_ settings and when adjusting these parameters according to patient response. APRV was used as a rescue measure before, during, and after the COVID-19 pandemic, using the same practice framework throughout the study period. Detailed APRV ventilator parameters could not be reliably collected due to inconsistent recording within the electronic clinical information system.

### 2.3. Outcomes

The primary outcome was improvement in mean oxygenation (P/F ratio) following APRV use after 6 h. Secondary outcomes included in-hospital mortality, ICU mortality, duration of mechanical ventilation, ICU length of stay, and effects on COVID-19 patients.

### 2.4. Statistical Analysis

Descriptive and outcome characteristics were reported as the median and interquartile ranges (IQR) for continuous variables and counts with percentages for categorical variables. Comparison of groups was made by Mann–Whitney U and Fisher’s exact test for continuous and categorical variables, respectively. The Kaplan–Meier estimator was used to describe overall survival and time to liberation from invasive mechanical ventilation. Patients were censored at the point of discharge from our intensive care units, and the log-rank test was used for comparison of APRV responders and non-responders. To describe the effect of APRV on physiological parameters (PO_2_, PCO_2_, and pH) across the cohort, an interrupted time series (ITS) model was constructed. We used a linear mixed-effects model, allowing random effects specified for physiological parameters (PO_2_, PCO_2_, and pH) while clustering at the participant level [25]. Variables with a *p*-value < 0.25 from univariable logistic regression for ICU mortality were included in a multivariable model. Subsequent backwards selection was performed using the Akaike Information Criterion (AIC) to produce a final model. All model assumptions were tested graphically. Analysis was conducted in R (version 4.5.0) using the nlme, survival, and MASS packages.

## 3. Results

### 3.1. Patient Characteristics

**Figure 1:**
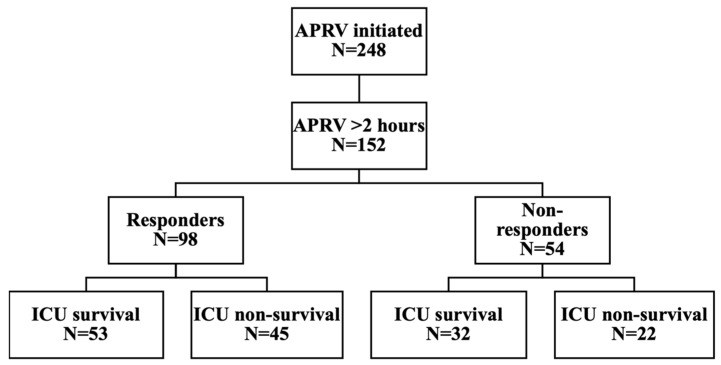
Flow chart—stratified according to responders (improvement in oxygenation (P/F ratio at 6 h) and non-responders (no improvement or worsening of P/F ratio at 6 h) with description of ICU mortality of patients included in the study.

A total of 248 patients had APRV initiated for AHRF, and of these, 152 met the inclusion criteria of being APRV ventilated for longer than 2 h (Figure 1). The median age was 62 years, and the patient cohort was predominantly male (64%). The median APACHE II and SOFA scores were 16 (Interquartile range (IQR) 13–23) and 11 (IQR 10–14), respectively. The median RALE score was 32, which reflects moderate to severe ARDS. The median Charlson comorbidity score (CCS) was 2 (IQR 1–4), with hypertension (25%), type 2 diabetes (19%), and asthma (12%) being common comorbidities across the population. COVID-19 was implicated in 44% of ICU discharge diagnoses.

We categorised the cohort as responders or non-responders based on the change in P/F ratio within 6 h of APRV initiation. Responders were defined as patients who showed any positive improvement in P/F ratio post-APRV during the 6 h period, whereas non-responders were patients with a decline or no improvement in oxygenation. Across the cohort, 98 patients (64%) were responders, and 54 (36%) were non-responders. There were no significant differences in the demographic variables between these groups (Table 1).

### 3.2. Outcome: Improvement in Oxygenation

**Figure 2:**
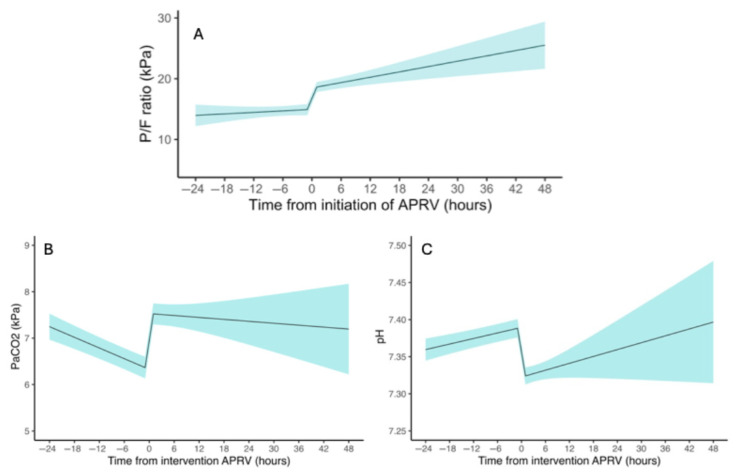
Interrupted time series model displaying the change in P/F ratio (**A**), PaCO_2_ (**B**), and pH (**C**) per hour prior to and following initiation of airway pressure release ventilation.

Interrupted time series (ITS) models were produced for P/F ratio, PCO_2_, and pH (Table 2; Figure 2) following graphical interpretation of the ITS model for the P/F ratio satisfaction of model assumptions. A total of 2884 samples of physiological parameters were included in our analysis, with a median of 20 samples (IQR 15–22) per patient. Missing observations were not imputed; instead, the mixed-effects modelling framework allowed the analysis of all available data points. At the point of APRV initiation, there was an increase in the P/F ratio of 3.54 kPa (95% CI 2.71, 4.36; *p* < 0.01) and PaCO_2_ of 1.24 kPa (95% CI 1.13–1.35; *p* < 0.01), with a decrease in pH of 0.07 (95% CI −0.07, −0.06; *p* < 0.01). After APRV, the trends in P/F ratio (*p* = 0.09) and pH (*p* = 0.76) did not differ from the pre-intervention trend. The pre-APRV decreasing trend in PaCO_2_ of −0.04 kPa per hour (95% CI −0.03 to −0.05; *p* < 0.01) was reversed by 0.04 kPa per hour (0.03; 95% CI 0.01–0.05; *p* < 0.01), resulting in no summative change in the post-APRV PaCO_2_ (−0.01 kPa; 95% CI −0.03 to 0.01; *p* = 0.51). This suggests that the intervention produces an immediate effect of oxygenation improvement at the expense of CO_2_ clearance.

The median lowest P/F ratio prior to APRV initiation was 9.9 (7.9–11.1) kPa. A total of 64.5% of patients had an improvement in P/F ratio 6 h after initiation of APRV. Prior to APRV, those in the responder category exhibited significantly greater hypoxemia, with a lower lowest P/F ratio prior to APRV (9.8, IQR 7.7–11.1 vs. 10.4, IQR 9.0–11.9 kPa, *p* = 0.046) and lower P/F ratio 6 h pre-APRV (12.3, IQR 10.2–14.5 vs. 14.4, IQR 12.1–17.5 kPa, *p* < 0.01). Responders also required a higher FiO_2_ (72.5%, IQR 60.0–85.0 vs. 67.5%, IQR 60–75, *p* = 0.03) and had a lower PaCO_2_ (6.4, IQR 5.7–7.2, vs. 7.1, IQR 6.0–8.0 kPa, *p* = 0.04). Following APRV initiation, responders demonstrated a significant improvement in oxygenation, with a median P/F ratio of 18.8 kPa (IQR 15.3–23.1) compared to 11.9 kPa (IQR 10.2–13.6) in non-responders (*p* < 0.01). Responders also had a higher PaO_2_ (10.1, IQR 9.2–10.7 vs. 9.1, IQR 8.3–9.6 kPa, *p* < 0.01) and required a lower FiO_2_ (57.5%, IQR 50.0–71.3 vs. 75%, IQR 65–80, *p* = 0.04). There were no significant differences in pH (*p* = 0.31) or PaCO_2_ (*p* = 0.09) between groups post-APRV (Table 3).

### 3.3. COVID-19 vs. Non-COVID-19 Patients with AHRF 

Of the 152 patients, 67 (44%) were COVID-19 positive and 85 (56%) were admitted due to non-COVID-19-related pneumonia. Age and sex distribution were similar between groups. Patients with COVID-19 had a higher BMI (30.8 vs. 26 kg/m^2^) and had increased comorbidities of type 2 diabetes mellitus (29.9% vs. 10.6%) and hypertension (34.3% vs. 17.6%). ICU admission severity scores were lower in the COVID-19 group (SOFA, 11 vs. 12 and APACHE II, 15 vs. 18), whereas RALE scores were comparable between both groups. COVID-19 patients were more frequently treated with glucocorticoids, anticoagulants, and immunosuppressants on admission compared to non-COVID-19 patients. The detailed description of these two groups is presented in Appendix A.1.

There was no difference in the lowest P/F ratio prior to APRV initiation between COVID-19 and non-COVID-19 patients (9.8, IQR 8.0–10.8 vs. 9.9, IQR 7.8–11.8, *p* = 0.77). Gas exchange parameters 6 h before APRV, including P/F ratio, FiO_2_, and PaO_2_, were similar between groups. Non-COVID-19 patients had a lower pH pre-APRV (*p* = 0.01). Six hours post-APRV initiation, oxygenation improved in both groups with the same median P/F ratios of 15.3 kPa and no between-group differences in FiO_2_, PaO_2_, or PaCO_2_ (all *p* > 0.2). pH remained lower in non-COVID-19 patients post-APRV (*p* = 0.02) (Table 4).

### 3.4. Other Outcomes

All other outcomes comparing responders and non-responders are detailed in Table 5. ICU mortality, (46% of responders vs. 41% of non-responders; *p* = 0.60), hospital mortality (48% of responders vs. 44% of non-responders; *p* = 0.70), length of ICU stay (12.4 days for responders vs. 15.7 days for non-responders, *p* = 0.60), and length of hospital stay (29 days for responders vs. 29.5 days for non-responders, *p* = 0.60) were similar between groups. The duration of ventilation was similar between those who responded to APRV for the total duration of mechanical ventilation (12.5 days for responders vs. 14.1 days for non-responders, *p* = 0.94), ventilation prior to APRV (0.7 for responders vs. 1.8 for non-responders, *p* = 0.13), and those who did not respond to APRV. Use of adjuvant therapies (prone positioning before/during APRV and inhaled nitric oxide) also did not differ significantly between the responder and non-responder groups (Table 5).

The outcome variables were also stratified according to COVID-19 status at ICU discharge. Mortality, ICU length of stay, and hospital length of stay were all similar between COVID-19 and non-COVID-19 patients, and use of adjuvant therapies (prone positioning before/during APRV and nitric oxide) was more frequent in COVID-19 patients (*p* ≤ 0.05). Full COVID-19 stratified outcomes are presented in the Supplementary Table S1.

### 3.5. Survival Analysis

**Figure 3:**
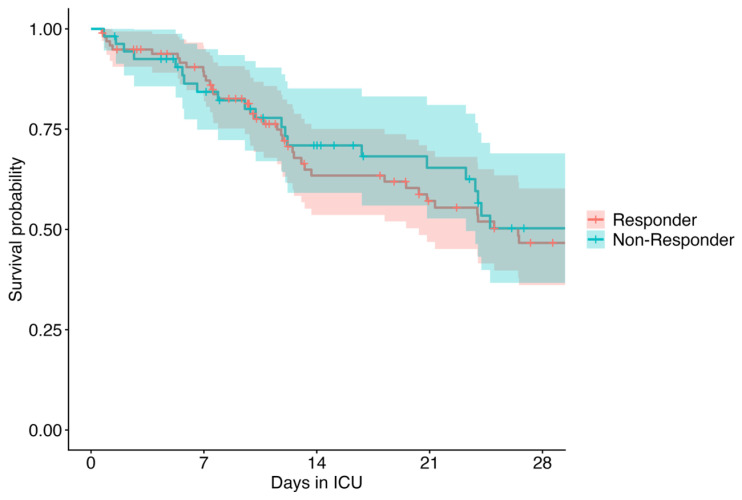
Kaplan–Meier curve for the probability of survival stratified according to the group’s responders and non-responders to airway pressure release ventilation. Patients were censored at the point of discharge from the intensive care unit.

Kaplan–Meier survival analysis was used to assess the probability of survival during admission to the ICU for responders and non-responders to APRV, with patients censored at ICU discharge. The response to APRV is not associated with short-term ICU survival in this cohort, and there was no significant difference in ICU survival between patients who responded and those who did not respond to airway pressure release ventilation (log-rank *p* = 0.48) (Figure 3).

Univariable logistic regression modelling was used to examine factors associated with ICU mortality, with results provided in the Appendix A.2 Table A2. Variables with a *p*-value < 0.25 from univariable logistic regression for ICU mortality were included in a multivariable model. Given its relevance to the study hypothesis, APRV response was also evaluated in the multivariable model using forced inclusion regardless of univariable significance. Subsequent backwards selection was performed using the Akaike Information Criterion (AIC) to produce a final model with ICU mortality as the dependent variable. Covariates included age, sex (male), medical history of asthma, Charlson Comorbidity Index, use of inhaled nitric oxide (NO), RALE score, and APACHE II score. After adjustment for the covariates, a medical history of asthma was the only variable associated with ICU mortality (OR 3.6, 95% CI 1.11–13.1). However, the wide confidence interval, which reflects the small number of patients with asthma in this cohort, limits statistical precision (Table 6).

### 3.6. APRV and Ventilation

**Figure 4:**
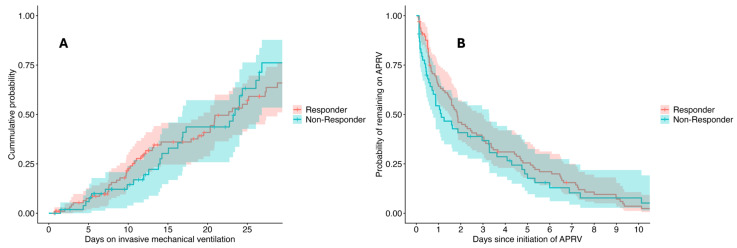
Kaplan–Meier for responders stratified by responders and non-responders to airway pressure release ventilation (APRV). Panel (**A**) is a cumulative probability of being liberated from invasive mechanical ventilation over time. Panel (**B**) is the probability of remaining on APRV over time. Patients were censored at the point of discharge from the intensive care unit.

Further time-to-event analysis for the cumulative probability of liberation from invasive mechanical ventilation over time was conducted (Figure 4). While responders and non-responders did not differ in the time from liberation from mechanical ventilation (log-rank *p* = 0.96), when comparing the probability of remaining on APRV over time, both groups show a rapid early decline. This indicates frequent early transition off APRV with no statistical difference between the groups (*p* = 0.60), suggesting that response status was not associated with time to liberation from ventilation or duration of APRV in this cohort.

## 4. Discussion

In our study, we identified 152 patients with AHRF who had APRV initiated as a rescue measure for more than two hours. Approximately two-thirds of patients (64.5%) showed improved oxygenation 6 h after the initiation of APRV. When comparing patients who had improved oxygenation to those who did not, there were no significant differences in ICU mortality rate or in ICU/hospital length of stay. The overall ICU and hospital mortality were 44% and 47%, respectively, reflecting the severity of the AHRF in these patients. All patients met ARDS criteria and had a median RALE score of 32, indicating severe radiological changes in combination with severe hypoxemia, likely to reflect severe ARDS phenotype. To our knowledge, this represents one of the largest cohorts to date evaluating APRV as a rescue intervention for acute severe AHRF.

We defined a “response” to APRV as an improvement in the mean P/F ratio within six hours of starting APRV compared to the six hours before its initiation. This definition was established to highlight that some patients do not show improvement in oxygenation, distinguishing them from those who do respond. Following the initiation of APRV, we observed a significant, abrupt improvement in oxygenation and changes in other ventilation indices, consistent with previous studies [15,26]. Log-transformed interrupted time series (ITS) modelling demonstrated an immediate increase in the P/F ratio of 3.54 kPa, along with a rise in PaCO_2_ and a transient reduction in pH. These findings are consistent with the known physiological mechanisms of APRV, which promote alveolar recruitment and improved mean airway pressure but may impair CO_2_ clearance due to shortened release time and changes in transpulmonary pressures [5].

Prior to the initiation of APRV, the responders exhibited more severe hypoxemia compared to non-responders—reflected by lower pre-intervention P/F ratio and PaO_2_ levels along with significantly higher FiO_2_ requirement, consistent with more severe AHRF (P/F < 13.3 kPa) [19]. The substantial post-APRV improvement in this group supports the idea that APRV may provide its most significant benefits to those with severe hypoxemia. However, patients with lower baseline P/F ratios may have greater potential for improvement following intervention, and therefore regression to the mean may partially contribute to the observed differences in oxygenation response. Consistent with previous findings, the improved oxygenation was not associated with better survival [16,17]. This outcome highlights an important distinction—improved oxygenation does not necessarily lead to overall benefits. Improvements in oxygenation are frequently used as physiological markers of treatment response in ARDS; however, changes in surrogate endpoints such as the P/F ratio may not necessarily translate into improved clinical outcomes. Multiple other factors, such as the underlying cause of respiratory failure, the patient’s demographics, such as age, gender, and comorbidity burden, and the degree of multisystem involvement, may overshadow the benefits achieved through ventilatory optimisation. Future research could help clarify whether alternative definitions of “response” or a longer evaluation period better predict outcomes.

The randomised trials of APRV to date have been very small and heterogeneous, with inconsistent interventions, and the meta-analysis of these trials shows conflicting results [26,27]. Moreover, a Canadian Health Technology Review published on APRV also concluded that there is no mortality benefit from the use of APRV in ARDS [17]. Most RCTs use APRV as an initial mode of ventilation in comparison to most published observational studies, which use APRV as a rescue strategy after failing conventional ventilation for severe AHRF [15]. According to a survey conducted in the UK, the use of APRV has become more common, but more as a rescue strategy [12,28]. A large multi-centre study is currently underway to evaluate APRV as a primary ventilation strategy, comparing it with conventional ventilation for moderate to severe AHRF [29].

Our cohort also consisted of patients with COVID-19 (44%). While there were differences in demographic variables between COVID-19 and non-COVID-19 patients (e.g., BMI, presence of diabetes mellitus and hypertension, SOFA and APACHE II scores), there were no significant differences in overall outcomes in mortality or ICU/Hospital length of stays. Moreover, there were no differences in oxygenation and gas exchange variables between the COVID-19 and non-COVID-19 groups. Also, there were no significant differences in RALE scores. The comparison between COVID-19 and non-COVID-19 patients was performed as an exploratory analysis and should therefore be interpreted with caution.

Our study has several limitations. Firstly, it is a single-centre observational study, which means that the findings may not be generalisable due to variations in practices and differing levels of experience with APRV. Secondly, this is a retrospective cohort, and although data were obtained from all available sources, there was limited, inconsistent information on preinitiation ventilation modes and settings, including APRV parameters (P_high_, P_low_, T_high_, T_low_, and PEEP), complications, and the reasons for discontinuation of APRV, which were inconsistently captured by the electronic platform. Consequently, we are unable to discuss the variables related to initiation and titration, nor their impact on outcomes. However, while mechanical factors contribute to respiratory failure in addition to impaired oxygenation, the P/F ratio remains a widely used clinical marker of oxygenation impairment in patients with acute hypoxemic respiratory failure and therefore represents a valid parameter for assessing physiological response. Moreover, we only included those with severe AHRF who needed APRV. As a result, confounding by indication cannot be excluded, as APRV may have been preferentially used in the sickest patients or at specific stages of respiratory failure. Also, the absence of a comparative control group with conventional ventilation limits direct comparisons with other ventilation strategies. However, despite these limitations, this is the largest rescue APRV cohort reported to date, detailing the granularity of oxygenation variables in relation to outcomes.

Our study expands the understanding of APRV use in the UK and supports the conclusion that APRV reliably improves oxygenation but may not necessarily alter mortality. A larger multi-centre dataset may help clarify selective enrichment groups who may benefit from APRV. Establishing standardised response criteria and uniform APRV initiation protocols will also be essential for advancing the evidence base. By refining patient selection and ventilation strategies, future research may clarify the precise role of APRV in the management of acute hypoxemic respiratory failure. The ongoing multicentre RELEASE Trial (ISRCTN17158033) is evaluating APRV versus conventional ventilation in moderate-to-severe acute hypoxaemic respiratory failure to determine its impact on ventilator duration, quality of life, and cost-effectiveness [29].

## 5. Conclusions

In this single-centre cohort, APRV was used primarily as a rescue ventilation strategy in patients with severe AHRF. While APRV use was associated with early improvements in oxygenation in approximately two-thirds of patients, this physiological response did not translate into improved clinical outcomes. Instead, the outcomes were driven by patient-level factors such as age and comorbidity burden, most significantly a medical history of asthma. These findings suggest that although APRV reliably improves oxygenation, its use as a rescue intervention may not sufficiently improve overall survival. Prospective randomised trials with standardised APRV protocols are needed to clarify which patient groups may benefit most, with the ongoing multi-centre RELEASE trial representing an important step toward addressing these questions.

## Figures and Tables

**Table 1:** Admission characteristics between patients who responded and did not respond to airway pressure response ventilation.

	All N = 152	Response N = 98	Non-Responders N = 54	*p* Value
Age	62 (53, 70)	63 (53, 72)	61 (53, 66)	0.20
Male	98 (64.5%)	65 (66.3%)	33 (61.1%)	0.60
BMI	28.4 (23.0, 34.8)	27.0 (22.6, 34.7)	29.4 (25.1, 34.8)	0.49
Comorbidity				
Charlson Comorbidity Index	2 (1, 4)	2 (1, 4)	2 (1, 4)	0.73
Asthma	18 (11.8%)	12 (12.2%)	6 (11.1%)	>0.99
Chronic obstructive pulmonary disease	6 (3.9%)	5 (5.1%)	1 (1.9%)	0.42
Type 2 diabetes mellitus	29 (19.1%)	18 (18.4%)	11 (20.4%)	0.83
Cardiovascular disease	41 (27.0%)	29 (29.6%)	12 (22.2%)	0.34
History of cancer	8 (5.3%)	5 (5.1%)	3 (5.6%)	>0.99
Radiology Score				
RALE score	32 (26, 38)	31 (24, 38)	32 (27, 37)	0.32
ICU Severity Scores				
SOFA score	11 (10, 14)	11 (10, 14)	11 (10, 14)	0.79
APACHE II score	16 (13, 23)	16 (13, 23)	17 (14, 22)	>0.99
Reason for admission				
Other Pneumonia	85 (55.9%)	52 (53.1%)	33 (61.1%)	>0.99
COVID-19	67 (44.1%)	46 (46.9%)	21 (38.9%)	0.40

Data are presented as median (interquartile range or number (%) as appropriate. *p*-values represent comparisons between responders and non-responders to APRV, assessing differences in baseline characteristics between groups using either the Mann–Whitney U test or Fisher’s exact test. Abbreviations: APACHE—acute physiological and chronic health evaluation; BMI—body mass index; COVID-19—coronavirus disease 2019; RALE—radiographic assessment of lung edema; SOFA—sequential organ failure assessment.

**Table 2:** Interrupted time series models for response to airway pressure release ventilation. Modelling of variables representing oxygenation (P/F ratio), ventilation (PaCO_2_), and acidosis (pH), described per hour.

Variable	P/F Ratio		PaCO_2_		pH	
	Change in kPa per Hour (95% CI)	*p* Value	Change in kPa per Hour (95% CI)	*p* Value	Change in pH per Hour (95% CI)	*p* Value
Intercept	15.0 (14.0, 15.9)	<0.01	6.3 (6.1, 6.6)	<0.01	7.4 (7.4–7.4)	<0.01
Pre-intervention	0.04 (−0.05, 1.1)	0.37	−0.04 (−0.03, −0.05)	<0.01	0.00 (0.00, 0.00)	<0.01
ARPV	3.5 (2.7, 4.4)	<0.01	1.2 (1.1, 1.4)	<0.01	−0.07 (−0.07, −0.06)	<0.01
Post-intervention difference	0.1 (−0.02, 0.23)	0.09	0.03 (0.01, 0.05)	0.01	0.00 (0.00, 0.00)	0.76

Abbreviations: P/F ratio—ratio of partial pressure of oxygen and fraction of inspired oxygen; PaCO_2_—partial pressure of arterial carbon dioxide; APRV—airway pressure release ventilation.

**Table 3:** Arterial blood gas results and oxygenation parameters before and after initiation of airway pressure release ventilation (APRV) between patients who responded and did not respond to APRV.

	All N = 152	Responders N = 98	Non-Responders N = 54	*p* Value
Lowest P/F ratio prior to APRV initiation				
P/F ratio (kPa)	9.9 (7.9, 11.1)	9.8 (7.7, 11.1)	10.4 (9.0, 11.9)	0.05
Blood gases 6 h prior to initiation of APRV				
P/F ratio (kPa)	13.1 (11.9, 17.1)	12.3 (10.2, 14.5)	14.4 (12.1, 17.5)	<0.01
FiO_2_ (%)	70.0 (60.0, 77.5)	72.5 (60.0, 85.0)	67.5 (60.0, 75.0)	0.03
PaO_2_ (kPa)	8.9 (8.2, 9.6)	8.8 (8.2, 10.0)	8.7 (8.1, 9.5)	0.20
PaCO_2_ (kPa)	6.8 (5.8, 7.7)	6.4 (5.7, 7.2)	7.1 (6.0, 8.0)	0.04
pH	7.4 (7.3, 7.4)	7.4 (7.3, 7.4)	7.4 (7.3, 7.4)	0.70
Blood gases 6 h post initiation of APRV				
P/F ratio (kPa)	14.0 (11.0, 16.8)	18.8 (15.3, 23.1)	11.9 (10.2, 13.6)	<0.01
FiO_2_ (%)	67.5 (57.5, 80.0)	57.5 (50.0, 71.3)	75.0 (65.0, 80.0)	0.04
PaO_2_ (kPa)	9.5 (8.4, 10.1)	10.1 (9.2, 10.7)	9.1 (8.3, 9.6)	<0.01
PaCO_2_ (Kpa)	7.7 (7.0, 8.7)	7.0 (6.0, 8.0)	8.2 (7.2, 8.7)	0.09
pH	7.3 (7.3, 7.4)	7.3 (7.3, 7.4)	7.3 (7.3, 7.4)	0.30

Data points are presented as median (interquartile range). *p*-values represent comparisons between responders and non-responders to APRV, assessing differences in oxygenation and ventilatory parameters at specified time points relevant to APRV initiation. Abbreviations: P/F ratio—ratio of partial pressure of oxygen in arterial blood (PaO_2_) to the fraction of inspired oxygen (FiO_2_); PaCO_2_—partial pressure of carbon dioxide in arterial blood; APRV—airway pressure release ventilation.

**Table 4:** Arterial blood gas results and oxygenation parameters before and after initiation of airway pressure release ventilation (APRV) between patients who were and were not diagnosed with COVID-19 on admission to ICU.

	All N = 152	COVID-19 N = 67	Non-COVID-19 N = 85	*p* Value
Lowest P/F ratio prior to APRV initiation				
P/F ratio (kPa)	9.9 (7.9, 11.1)	9.8 (8.0, 10.8)	9.9 (7.8, 11.8)	0.77
Blood gases 6 h prior to initiation of APRV				
P/F ratio (kPa)	13.8 (11.4, 17.5)	13.7 (11.4, 17.7)	13.9 (11.5, 17.2)	0.95
FiO_2_ (%)	65.0 (55.0, 77.5)	65.0 (52.5, 80.0)	65.0 (57.5, 75.0)	0.87
PaO_2_ (kPa)	8.9 (7.9, 9.9)	9.1 (7.9, 9.9)	8.8 (7.8, 10.2)	0.94
PaCO_2_ (kPa)	6.3 (5.2, 7.4)	6.2 (4.9, 7.2)	6.6 (5.7, 7.5)	0.06
pH	7.4 (7.3, 7.5)	7.4 (7.4, 7.5)	7.4 (7.3, 7.4)	0.01
Blood gases 6 h post initiation of APRV				
P/F ratio (kPa)	15.3 (12.3, 20.8)	15.3 (12.3, 21.1)	15.3 (12.6, 20.2)	0.98
FiO_2_ (%)	68.3 (56.7, 81.7)	70.0 (56.7, 85.0)	66.7 (56.7, 78.3)	0.24
PaO_2_ (kPa)	10.0 (8.9, 11.6)	10.0 (9.0, 11.7)	10.0 (8.9, 11.4)	0.63
PaCO_2_ (Kpa)	7.5 (6.5, 8.7)	7.5 (6.5, 8.6)	7.5 (6.6, 8.7)	0.48
pH	7.3 (7.3, 7.4)	7.3 (7.3, 7.4)	7.3 (7.3, 7.4)	0.02

Data are presented as median (interquartile range); *p*-values represent comparisons between patients with and without COVID-19, assessing differences in oxygenation and ventilatory parameters at specified time points relevant to APRV initiation. Abbreviations: P/F ratio—ratio of partial pressure of oxygen in arterial blood (PaO_2_) to the fraction of inspired oxygen (FiO_2_); PaCO_2_—partial pressure of carbon dioxide in arterial blood; APRV—airway pressure release ventilation.

**Table 5:** Comparison of clinical outcomes and use of adjunctive respiratory therapies for patients who responded and did not respond to airway pressure release ventilation.

Outcomes	All N = 152	Responders N = 98	Non-Responders N = 54	*p* Value
Mortality				
Intensive care	67 (44.1%)	45 (45.9%)	22 (40.7%)	0.60
Hospital	71 (46.7%)	47 (48.0%)	24 (44.4%)	0.70
Length of stay (days)				
Intensive care	13.2 (7.6, 29.7)	12.4 (7.6, 28.6)	15.67 (7.1, 31.0)	0.60
Hospital	29.0 (17.0, 62.0)	29.0 (19.0, 64.0)	29.5 (16.0, 55.0)	0.60
Duration of ventilation (days)				
Total mechanical ventilation	13.1 (7.6, 24.0)	12.5 (7.6, 24.0)	14.1 (7.1, 24.0)	0.94
Mechanical ventilation prior to APRV initiation	0.9 (0.1, 4.6)	0.7 (0.1, 4.3)	1.8 (0.1, 5.3)	0.13
Time to initiation of APRV from hospital admission	3.5 (0.95, 6.51)	3.2 (0.78, 6.58)	4.3 (1.99, 5.85)	0.15
Adjuvant respiratory therapy				
Prone position prior to APRV	87 (57.2%)	55 (56.1%)	32 (59.3%)	0.73
Prone positioning during APRV	89 (58.6%)	58 (59.2%)	31 (57.4%)	0.86
Nitric oxide therapy	31 (20.4%)	16 (16.3%)	15 (27.8%)	0.14

Data are presented as median (interquartile range) or number (%) as appropriate. *p*-values represent comparisons between responders and non-responders to APRV, assessing differences in clinical outcomes and use of adjunctive respiratory therapies between groups. Abbreviation: APRV—airway pressure release ventilation.

**Table 6:** Multivariable logistic regression model for backwards step elimination.

Characteristic	OR	95% CI	*p*-Value
Age	1.03	0.99, 1.08	0.11
Male	1.97	0.89, 4.49	0.10
Presence of Asthma	3.63	1.11, 13.1	0.04
Charlson Comorbidity Index	1.23	0.97, 1.60	0.11
Use of NO	2.10	0.83, 5.45	0.12
RALE score	1.04	1.00, 1.10	0.07
APACHE II score	1.05	0.99, 1.12	0.10
APRV Response	1.24	0.57, 2.73	0.60

Abbreviations: CI—confidence interval; OR—odds ratio; NO—nitric oxide.

## Data Availability

The original contributions presented in this study are included in the Supplementary Material reposited (link: https://doi.org/10.5258/SOTON/D3845). Further inquiries can be directed to the corresponding author.

## Supplementary Materials

The following supporting information can be downloaded at: https://www.mdpi.com/article/10.3390/jcm15072668/s1, Table S1: Full COVID-19 stratified outcomes.

## Abbreviations

The following abbreviations are used in this manuscript:

AHRF	Acute hypoxemic respiratory failure
AIC	Akaike Information Criterion
APACHE II	Acute Physiological and Chronic Healthy Evaluation II
APRV	Airway pressure release ventilation
ARDS	Acute respiratory distress syndrome
BMI	Body mass index
CCI	Charlson Comorbidity Index
CHARTS	Clinical Hospital Automated Record Tracking System
CI	Confidence interval
COPD	Chronic obstructive pulmonary disease
COVID19	Coronavirus disease 2019
CXR	Chest X-ray
FiO_2_	Fraction of inspired oxygen
HCRW	Health and Care Research Wales
HRA	Health Research Authority
IBW	Ideal body weight
ICNARC	Intensive Care National Audit and Research Centre
ICU	Intensive care unit
IMV	Invasive mechanical ventilation
IQR	Interquartile range
IRAS	Integrated Research Application System
ITS	Interrupted time series
NO	Nitric oxide
NHS	National Health Service
OR	Odds ratio
PaCO_2_	Partial pressure of arterial carbon dioxide
PaO_2_	Partial pressure of arterial oxygen
PCR	Polymerase chain reaction
PEEP	Positive end-expiratory pressure
P_high_	Pressure high
P_low_	Pressure low
P/F	PaO_2_/FiO_2_ ratio
RALE	Radiographic Assessment of Lung Edema
RCT	Randomised control trial
SOFA	Sequential Organ Failure Assessment
T_high_	Time at high pressure
T_low_	Time at low pressure
UK	United Kingdom

## Appendix A

### Appendix A.1

**Table A1:** Admission characteristics of patients who received airway pressure release ventilation categorised by COVID-19 status on ICU admission.

Demographics		COVID-19 Infection		
	All N = 152	Positive N = 67	Negative N = 85	*p* Value
Age	62 (53, 70)	62 (53, 70)	62 (53, 69)	0.77
Male	98 (64.5%)	46 (68.7%)	52 (61.2%)	0.39
BMI	28.4 (23.0, 34.8)	30.8 (25.8, 38.2)	26.0 (18.0, 31.1)	<0.01
Comorbidity				
Charlson Comorbidity Index	2.0 (1.0, 4.00)	3.0 (1.0, 4.0)	2.0 (1.0, 4.0)	0.33
Asthma	18 (11.8%)	11 (16.4%)	7 (8.2%)	0.14
Chronic obstructive pulmonary disease	6 (3.9%)	2 (3.0%)	4 (4.7%)	0.69
Type 2 diabetes mellitus	29 (19.1%)	20 (29.9%)	9 (10.6%)	<0.01
Ischaemic heart disease	3 (2.0%)	1 (1.5%)	2 (2.4%)	>0.99
Hypertension	38 (25.0%)	23 (34.3%)	15 (17.6%)	0.02
History of cancer	8 (5.3%)	3 (4.5%)	5 (5.9%)	>0.99
RALE score	32 (26, 38)	32 (26, 38)	32 (24, 38)	0.51
SOFA	11 (10, 14)	11 (10, 12)	12 (10, 15)	<0.01
APACHE II	16 (14, 23)	15 (13, 19)	18 (15, 25)	<0.01
Medications on admission				
Anti-coagulation	25 (16.4%)	18 (26.9%)	7 (8.2%)	<0.01
Glucocorticoids	50 (32.9%)	37 (55.2%)	13 (15.3%)	<0.01
Non-glucocorticoid immunosuppressant	12 (7.9%)	10 (14.9%)	2 (2.4%)	0.01
Nebulised medication	4 (2.6%)	4 (6.0%)	0 (0.0%)	0.04

### Appendix A.2

**Table A2:** Univariable logistic regression for ICU mortality.

Characteristic	OR	95% CI	*p*-Value
Age	1.06	1.03, 1.10	<0.001
Male	2.00	1.01, 4.05	0.05
Asthma	2.19	0.81, 6.28	0.12
COPD	2.63	0.50, 19.4	0.30
Type 2 diabetes mellitus	1.23	0.54, 2.79	0.60
Ischaemic heart disease	2.58	0.24, 56.3	0.40
Active cancer in previous year	2.20	0.52, 11.1	0.30
Nebulised asthma drugs	1.28	0.15, 10.9	0.80
Surgical injury	0.63	0.03, 6.70	0.70
Charlson Comorbidity Index	1.45	1.21, 1.78	<0.001
RALE score	1.04	1.00, 1.09	0.04
APACHE II score	1.05	1.00, 1.10	0.07
Response to APRV	1.23	0.63, 2.44	0.50
Nitric oxide therapy	2.03	0.92, 4.61	0.08
Prone position during APRV	0.87	0.46, 1.68	0.70
Prone position prior to APRV	0.96	0.50, 1.84	>0.90

## References

[1] Jivraj N.K., Hill A.D., Shieh M.S., Hua M., Gershengorn H.B., Ferrando-Vivas P., Harrison D., Rowan K., Lindenauer P.K., Wunsch H. (2023). Use of Mechanical Ventilation Across 3 Countries. JAMA Intern. Med..

[2] Case Mix Programme Public Report 2023–2024 Intensive Care National Audit & Research Centre 2023–2024. https://www.icnarc.org/wp-content/uploads/2025/06/CMP-Public-Report-2023-24-Infographic-Public.pdf?utm_.

[3] Brower R.G., Matthay M.A., Morris A., Schoenfeld D., Thompson B.T., Wheeler A., Acute Respiratory Distress Syndrome Network (2000). Ventilation with Lower Tidal Volumes as Compared with Traditional Tidal Volumes for Acute Lung Injury and the Acute Respiratory Distress Syndrome. N. Engl. J. Med..

[4] Dushianthan A., Cusack R., Chee N., Dunn J.O., Grocott M.P. (2014). Perceptions of diagnosis and management of patients with acute respiratory distress syndrome: A survey of United Kingdom intensive care physicians. BMC Anesthesiol..

[5] Swindin J., Sampson C., Howatson A. (2020). Airway pressure release ventilation. BJA Educ..

[6] Zhong X., Wu Q., Yang H., Dong W., Wang B., Zhang Z., Liang G. (2020). Airway pressure release ventilation versus low tidal volume ventilation for patients with acute respiratory distress syndrome/acute lung injury: A meta-analysis of randomized clinical trials. Ann. Transl. Med..

[7] Carsetti A., Damiani E., Domizi R., Scorcella C., Pantanetti S., Falcetta S., Donati A., Adrario E. (2019). Airway pressure release ventilation during acute hypoxemic respiratory failure: A systematic review and meta-analysis of randomized controlled trials. Ann. Intensive Care.

[8] Fredericks A.S., Bunker M.P., Gliga L.A., Ebeling C.G., Ringqvist J.R., Heravi H., Manley J., Valladares J., Romito B.T. (2020). Airway Pressure Release Ventilation: A Review of the Evidence, Theoretical Benefits, and Alternative Titration Strategies. Clin. Med. Insights Circ. Respir. Pulm. Med..

[9] Andrews P., Shiber J., Madden M., Nieman G.F., Camporota L., Habashi N.M. (2022). Myths and Misconceptions of Airway Pressure Release Ventilation: Getting Past the Noise and on to the Signal. Front. Physiol..

[10] Grasselli G., Calfee C.S., Camporota L., Poole D., Amato M.B.P., Antonelli M., Arabi Y.M., Baroncelli F., Beitler J.R., Bellani G. (2023). ESICM guidelines on acute respiratory distress syndrome: Definition, phenotyping and respiratory support strategies. Intensive Care Med..

[11] (2021). FICM/ICS ARDS Guideline (UK). https://www.ficm.ac.uk/sites/ficm/files/documents/2021-10/Guidelines_on_the_Management_of_Acute_Respiratory_Distress_Syndrome.pdf.

[12] Ward J., Terrington I., Preston K., Smith A., Roe T., Barnes J., Allen E., Lima S., Cusack R., Grocott M.P. (2025). Management of adult mechanically ventilated patients: A UK-wide survey. J. Intensive Care Soc..

[13] Dushianthan A., Cumpstey A.F., Ferrari M., Thomas W., Moonesinghe R.S., Summers C., Montgomery H., Grocott M.P. (2022). Intensive care physicians’ perceptions of the diagnosis & management of patients with acute hypoxic respiratory failure associated with COVID-19: A UK based survey. J. Intensive Care Soc..

[14] Ibarra-Estrada M.A., Garcia-Salas Y., Mireles-Cabodevila E., Lopez-Pulgarin J.A., Chavez-Pena Q., Garcia-Salcido R., Mijangos-Méndez J.C., Aguirre-Avalos G. (2022). Use of Airway Pressure Release Ventilation in Patients With Acute Respiratory Failure Due to COVID-19: Results of a Single-Center Randomized Controlled Trial. Crit. Care Med..

[15] Mahmoud O., Patadia D., Salonia J. (2021). Utilization of Airway Pressure Release Ventilation as a Rescue Strategy in COVID-19 Patients: A Retrospective Analysis. J. Intensive Care Med..

[16] Roshdy A., Elsayed A.S., Saleh A.S. (2023). Airway Pressure Release Ventilation for Acute Respiratory Failure Due to Coronavirus Disease 2019: A Systematic Review and Meta-Analysis. J Intensive Care Med..

[17] CADTH Heatlh Technology Review. https://www.canjhealthtechnol.ca/index.php/cjht/article/download/RC1451/914?inline=1.

[18] von Elm E., Altman D.G., Egger M., Pocock S.J., Gøtzsche P.C., Vandenbroucke J.P., STROBE Initiative (2007). Strengthening the Reporting of Observational Studies in Epidemiology (STROBE) statement: Guidelines for reporting observational studies. BMJ.

[19] Ranieri V.M., Rubenfeld G.D., Thompson B.T., Ferguson N.D., Caldwell E., Fan E., Camporota L., Slutsky A.S., ARDS Definition Task Force (2012). Acute respiratory distress syndrome: The Berlin Definition. JAMA.

[20] Charlson M.E., Pompei P., Ales K.L., MacKenzie C.R. (1987). A new method of classifying prognostic comorbidity in longitudinal studies: Development and validation. J. Chronic Dis..

[21] Knaus W.A., Draper E.A., Wagner D.P., Zimmerman J.E. (1985). APACHE II: A severity of disease classification system. Crit. Care Med..

[22] Vincent J.L., Moreno R., Takala J., Willatts S., De Mendonca A., Bruining H., Reinhart C.K., Suter P.M., Thijs L.G. (1996). The SOFA (Sepsis-related Organ Failure Assessment) score to describe organ dysfunction/failure. On behalf of the Working Group on Sepsis-Related Problems of the European Society of Intensive Care Medicine. Intensive Care Med..

[23] Warren M.A., Zhao Z., Koyama T., Bastarache J.A., Shaver C.M., Semler M.W., Rice T.W., Matthay M.A., Calfee C.S., Ware L.B. (2018). Severity scoring of lung oedema on the chest radiograph is associated with clinical outcomes in ARDS. Thorax.

[24] Zhou Y., Jin X., Lv Y., Wang P., Yang Y., Liang G., Wang B., Kang Y. (2017). Early application of airway pressure release ventilation may reduce the duration of mechanical ventilation in acute respiratory distress syndrome. Intensive Care Med..

[25] Saeed S., Moodie E.E.M., Strumpf E.C., Klein M.B. (2018). Segmented generalized mixed effect models to evaluate health outcomes. Int. J. Public Health.

[26] Othman F., Alsagami N., Alharbi R., Almuammer Y., Alshahrani S., Ismaeil T. (2021). The efficacy of airway pressure release ventilation in acute respiratory distress syndrome adult patients: A meta-analysis of clinical trials. Ann. Thorac. Med..

[27] Lim J., Litton E. (2019). Airway Pressure Release Ventilation in Adult Patients With Acute Hypoxemic Respiratory Failure: A Systematic Review and Meta-Analysis. Crit. Care Med..

[28] Rose L., Camporota L., Mills G.H., Laffey J., Perkins G.D., Shankar-Hari M., Szakmany T., McAuley D., RELEASE Investigators (2023). Airway pressure release ventilation: A survey of UK practice. Br. J. Anaesth..

[29] Does Setting the Breathing Machine (Ventilator) to Deliver Breaths Using a Method Called Airway Pressure Release Ventilation Help Patients with Diseased Lungs to Heal Faster and Spend Less Time on a Ventilator?. https://www.isrctn.com/ISRCTN17158033.

