# Supplementary material for: Outcome and Oxygenation Response to Airway Pressure Release Ventilation (APRV) Used as a Rescue Strategy for Severe Hypoxemic Respiratory Failure—An Observational Study
Source: J Clin Med. 2026 Apr 1;15(7):2668. doi: 10.3390/jcm15072668 (PMC13073732; doi:10.3390/jcm15072668)
Supplement: Supplementary file 1 [file jcm-15-02668-s001.zip › jcm-4172348-supplementary.pdf]

Supplementary Table S1. Full COVID-19 stratified outcomes.

Outcomes		COVID-19 infection		
	All N=152	Positive N=67	Negative N=85	P value
**Mortality**				
Intensive care	67 (44.1%)	30 (44.8%)	37 (43.5%)	>0.99
Hospital admission	71 (46.7%)	32 (47.8%)	39 (45.9%)	0.87
**Length of stay (days)**				
Intensive care	13.2 (7.6, 29.7)	13.2 (10.0, 26.5)	13.3 (6.4, 32.9)	0.31
Hospital admission	29.0 (17.0, 62.0)	33.0 (20.0, 60.0)	28.0 (16.0, 64.0)	0.24
**Duration of ventilation (days)**				
Total mechanical ventilation	13.1 (7.6, 24.0)	13.0 (10.1, 24.4)	13.3 (6.4, 23.9)	0.14
Mechanical ventilation prior to APRV initiation	0.9 (0.1, 4.6)	0.7 (0.0, 4.3)	1.3 (0.1, 5.0)	0.11
Time to initiation of APRV	3.5 (1.0, 6.5)	3.5 (1.0, 6.4)	3.6 (0.8, 6.6)	0.83
**Adjuvant respiratory therapy**				
Prone position prior to APRV	87 (57.2%)	50 (74.6%)	37 (43.5%)	<0.01
Prone positioning during APRV	89 (58.6%)	49 (73.1%)	40 (47.1%)	<0.01
Nitric oxide therapy	31 (20.4%)	19 (28.4%)	12 (14.1%)	0.04
